# Laser-induced breakdown spectroscopy (LIBS) as a novel technique for detecting bacterial infection in insects

**DOI:** 10.1038/s41598-019-39164-8

**Published:** 2019-02-21

**Authors:** Nabil Killiny, Ed Etxeberria, Alejandro Ponce Flores, Pedro Gonzalez Blanco, Teresa Flores Reyes, Luis Ponce Cabrera

**Affiliations:** 1 University of Florida, Citrus Research and Education Center, 700 Experiment Station Road, Lake Alfred, FL USA; 2 Universidad Nacional Autonoma de Mexico, Fac. De Ciencias, Universidad 3000, Circuito Exterior S/N, Distrito Federal, 04510 Mexico; 3 Instituto Politecnico Nacional, CICATA, Carretera Tampico-Puerto Industrial Altamira Km 14.5, Industrial Altamira, 89600 Altamira, Tampico Mexico

## Abstract

To prevent the spread of diseases in humans, animals or plants, determining whether potential vectors are infected is crucial. For example, early detection of the citrus disease Huanglongbing, which has been a scourge on the citrus industries around the world, is a critical need. This vector-borne disease is transmitted by *Diaphorina citri*, the Asian citrus psyllid, which carries the putative bacterial phytopathogen, *Candidatus* Liberibacter asiaticus (*C*Las). In this investigation, we introduced Laser-Induced Breakdown Spectroscopy (LIBS) to reveal key biochemical differences between *C*Las-infected and non-infected psyllids. The emission spectra captured from laser ablation of *C*Las-infected and healthy psyllids were processed through the principal component analysis (PCA) method and compared. Thirteen peaks from seven different elements were detected in *D*. *citri*. The *t*-test showed that *C*Las-infected *D*. *citri* were deficients in zinc, iron, copper, magnesium, calcium, and nitrogen. The PCA showed that LIBS can successfully differentiate between *C*Las-infected and healthy *D*. *citri* by comparing their elemental profile. In this work, we demonstrated a method that allows for a fast and precise compositional microanalysis of an insect vector which can contribute to the early detection of citrus huanglongbing

## Introduction

Huanglongbing (HLB), or citrus greening disease, has been a scourge on citrus industries around the world. The vector-borne disease is transmitted by two species of psyllids (Hemiptera: Liviidae): the Asian citrus psyllid, *Diaphorina citri*, and the African psyllid, *Trioza erytrea*^1,2^. Like other Hemiptera, psyllids are phloem sap feeders. These two species feed and reproduce on all members of the family Rutacea, which includes all citrus species of commercial importance. During the feeding process, the psyllid transmits the putative phytopathogen *Candidatus* Liberibacter asiaticus (*C*Las), a Gram-negative alpha-proteobacterium^3,4^. Currently, *C*Las remains unculturable complicating research efforts. In addition to *C*Las, psyllids host several other bacterial endosymbionts in their gut^5^.

In Florida, HLB is widespread, already affecting almost 100% of citrus groves^6–8^. However, in states such as Texas, Arizona, California, Mediterranean area and Australia, disease presence is much lower, making early detection a priority. For several reasons, detecting HLB in citrus trees is difficult at best. First, there is a six- to nine-month asymptomatic period after inoculation^9,10^. During this time, the trees appear healthy, whilst feeding psyllids can acquire and spread the bacterium from tree to tree. Foliar symptoms include blotchy mottling (uneven distribution of chlorophyll in the leaves), vein corking, starch accumulation, and leaf chlorosis, which resembles zinc deficiency^11–13^. Symptoms in fruit include small size, lopsided fruit, aborted seeds, bitter off-flavors, early fruit drop, and uneven coloration during ripening. Once trees are heavily symptomatic, tree death usually occurs within five years, depending on their variety and tolerance to HLB. Second, some citrus cultivars are more tolerant to HLB than others^14–16^. In Florida, sweet oranges and grapefruits, which make up the majority of production, are particularly sensitive to HLB, while those of mandarin heritage are slightly more tolerant. Finally, the bacterial titer (a measurement of bacterial concentration) is not uniform within the tree, so a random sampling of leaves using conventional or quantitative polymerase chain reaction (PCR) methods may not reveal a presence of the bacterium^17^. Furthermore, low titers of *C*Las may not be detected by conventional PCR.

In uninfected citrus growing areas, the presence of psyllids is reported well before visual symptoms of HLB appear in trees. The primary method of psyllid control thus far has been insecticide application, but increased use of pesticides has resulted in resistance in some populations of *D*. *citri*^18^. Other methods to control *D*. *citri* include releasing the parasitic wasp *Tamarixia radiata*^19^ and using RNAi approaches^20–22^, although the latter has not been approved for commercial purposes. In HLB-affected areas, many growers have replaced dead or dying trees to reduce sources of inoculum. However, psyllids are still present in Florida, and they continue to actively transmit HLB. Early detection of *C*Las remains a critical goal and would be advantageous to both researchers and growers. Many PCR-based methods have been developed for detecting *C*Las in psyllids^23,24^. However, developing an effective, accurate, and inexpensive method is required to enable earlier disease detection. In areas where Asian citrus psyllid exists but the symptoms have not yet appeared on citrus trees, early detection of *C*Las-infected psyllids would be valuable.

Laser-induced breakdown spectroscopy (LIBS) technique offers many advantages for elements analysis^25–27^. It has gained a great popularity in elemental analysis because of its portability, lightning speed, low cost, nonrequirement for chemicals, minimal or no sample preparation, simultaneous determination of multiple elements, and capability to perform express identification^25–27^. The technique involves short, high-intensity laser pulses capable of ablating a small amount of material, thereby creating a momentary plasma. An optical fiber collects a portion of the light emitted from the plasma and delivers it to a spectrometer. The captured spectra are considered a “fingerprint” associated with a sample’s elemental composition.

In the last few years, LIBS has been used to study the effects of *C*Las infection on the nutritional composition of citrus plants^28,29^. It was recently demonstrated that LIBS can successfully differentiate between *C*Las-infected and healthy citrus plants by analyzing the major macro- and micronutrients^28^. LIBS analysis showed that *C*Las significantly decreased the level of calcium, magnesium, and potassium in citrus plants^29^. Recently, it has also been shown that combination of LIBS and Raman spectroscopy significantly improves discrimination and classification of bacterial species and strains^30^.

In this work, we introduce the LIBS technique for composition microanalysis of *D*. *citri*, the vector of citrus huanglongbing. LIBS can reveal the biochemical differences between *C*Las-infected and non-infected Asian citrus psyllids for immediate detection of the pathogen. To our knowledge, this is the first time that a LIBS technique has been directly applied to differentiate between pathogen-infected and pathogen-free vectors.

## Material and Methods

### Asian citrus psyllid colonies

*D*. *citri* colonies were continuously reared at the Citrus Research and Education Center, University of Florida (CREC-IFAS, UF, Lake Alfred, United States). Healthy psyllids were maintained on *C*Las-free alemow trees (*Citrus macrophylla*) in a USDA-APHIS/CDC-approved secured growth room (27 ± 1 °C, 65 ± 2% relative humidity, L16:D8 h photocycle). Monthly, random samples of *D*. *citri* adults and citrus leaves were collected and tested using polymerase chain reaction (PCR) assay as previously described^31^ to confirm that the plants remained *C*Las-free and the insects did not harbor *C*Las. *C*Las-infected psyllids colonies were reared on HLB-symptomatic and PCR-positive/*C*Las-infected *C*. *macrophylla* plants and maintained in the same conditions as described above. The *C*Las-infection rate was tested simultaneously (50 adult individuals per monthly sampling). *C*Las-infected and uninfected *D*. *citri* colonies were maintained in separate, USDA-APHIS/CDC-approved secured growth rooms to minimize the chance of cross-contamination. Mature adults (2–3 mm) were collected using an aspirator for LIBS assays.

### Laser-induced breakdown spectroscopy

We developed a compositional microanalysis procedure that allows us to obtain the elemental emission spectrum of an insect vector. For the analysis, we used a LIBS instrument, “SLIT-LIBS,” supplied by Onteko LLC (Tampa, United States), which includes a laser that emits in a “burst mode” regime described below. In this device, the laser beam is coupled with the optical path of a slit-lamp microscope for better visualization of samples.

**Figure 1:**
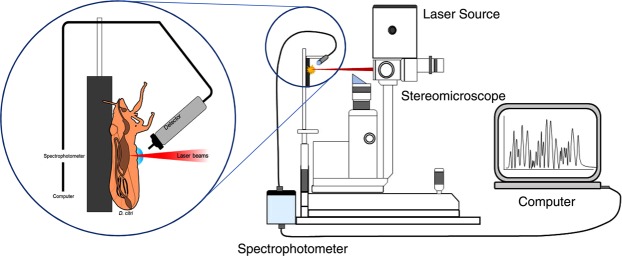
Schematic representation of laser-induced breakdown spectroscopy system used in this study. A stereomicroscope was modified and attached to the laser source to point laser beams on the abdomen of Asian citrus psyllid.

A schematic representation for this setup is shown in Fig. 1. The pulsed (neodymium: yttrium aluminum garnet) Nd:YAG laser emits at a wavelength of 1,064 nm while working in a Q-switch regime, producing light pulses (shots) with energy of up to 40 mJ at a repetition rate of 1 Hz. A low-power red laser was used to point where the Nd:YAG laser would impact and ablate the sample and generate the plasma. Each laser shot consisted of a train of three micropulses, each having a duration of 8 ns and an interval of 10–25 μs between them, resulting in an overall shot duration of about 70–80 μs. The laser beam was focused using a 50 mm focal length lens which produced a 40 µm diameter target on the samples. The laser ablation process induced the emission of light which was collected by an optical fiber and delivered to a cross Czerny–Turner spectrometer with a linear CCD as a detector. The spectral resolution of the system is 0.3 nm with a spectral range of 250–800 nm.

### Spectrum recording, data processing, and statistical analysis

We used adult Asian citrus psyllids from healthy (22 insects) and *C*Las-infected colonies (38 insects), the latter with an infection rate of 60% for LIBS detection of HLB. After spectra measurements, PCR was performed on all sampled psyllids as described previously^31^. PCR negatives from infected colonies were excluded from the statistical analysis. Consequently, only 22 *C*Las-infected psyllids were included in the statistical analysis. To obtain the spectra, psyllids were fixed with a double-sided-tape strip to a microscope slide held on a stand. Because *C*Las grows and multiplies in the insect’s haemolymph, the first laser pulse was required to perforate the exoskeleton in order to gain access to the interior of the psyllid. When the hemolymph exuded from the psyllid, a second laser pulse was delivered to ablate the exposed hemolymph and capture it’s spectrum. In total, four shots were performed on each insect: the first one to puncture the exoskeleton and release the haemolymph, and the others to calculate the average. The spectrometer was connected to a computer and spectra were stored using the SpectraSuite software (Ocean Optics, Tampa, United States). The average spectra of each sample was analyzed independently using the elemental database of the National Institute of Standards and Technology (NIST)^32^ and the LIBS Army elemental database^33^. The data were normalized by dividing the intensity of individual emission line by the total intensity of the total spectrum (i.e., the sum of the thirteen intensities). Statistical analyses were performed using JMP version 9.0 (SAS Institute Inc.). Principal component analysis (PCA) was performed using normalized data captured from the thirteen spectral lines. In addition, *t*-test (*p* < 0.05) was used to compare the level of each spectral line (normalized intensity) in *C*Las-infected *D*. *citri* with that of the controls. The PCA was repeated using only five of the captured spectral lines (Mg I, N II, CaO, Fe I, and CaOH), which were dramatically affected by *C*Las infection as shown by the *t*-test.

## Results

Thirteen peaks representing seven different elements were identified in the haemolymph of *D*. *citri* using LIBS (Table 1).

**Table 1:** Spectra lines detected and identified in the haemolymph of *D*. *citri* using LIBS.

Element or compound	Wavelength (nm)
Mg I	279.5
Fe II	298.9
Zn I	334.6
CN band	386.1–388.3
Cu I	406.2
Ca I	435.3
Ca I	462.1
N I	499.9
C-C	516.2
CaO band	547–556^44^
Fe I	566.3
CaOH	610.2^44^
H I	656.2

**Figure 2:**
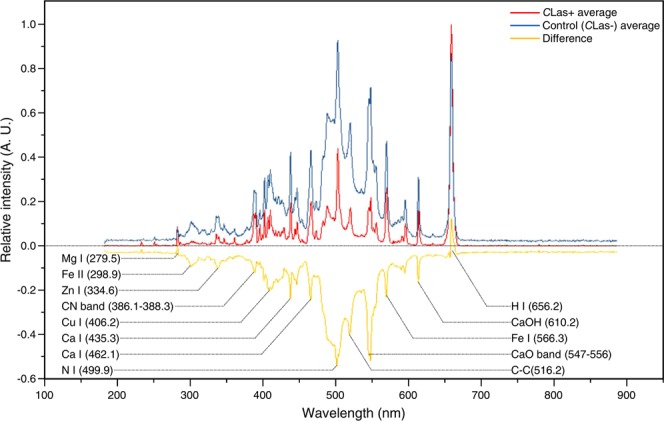
Typical spectra of healthy and *C*Las-infected Asian citrus psyllid (PCR-positive). Yellow spectrum is a subtraction between healthy and *C*Las-infected psyllids spectra.

A typical spectrum of healthy (blue) and *C*Las-infected (red) *D*. *citri* obtained with the LIBS after normalization is shown in Fig. 2. The wavelength of each detected peak is displayed in Fig. 2. One peak was identified for magnesium (279.5 nm), two peaks for iron (298.9, and 566.3 nm), one peak for zinc (334.6 nm), one for copper (406.2 nm), two for calcium (435.3, 462.1 nm), two for calcium-related compounds (CaO: 547–556 nm, CaOH: 610.2 nm), two for nitrogen (386.1–388.3 and 499.9 nm) or nitrogen-related compounds, and one for hydrogen (656.2 nm). Only *C*Las-infected psyllids which were confirmed positive using PCR were included in the data analysis. In the same manner, all control psyllids were also confirmed negative by PCR. In general, the peak intensity of most of the detected peaks was lower in *C*Las-infected *D*. *citri* Fig. 2. These included N II, CaO, Zn I, CaOH, Fe II, Ca I, and Cu I. The differences between the healthy and *C*Las-infected spectra are also shown in yellow (Fig. 2).

### LIBS successfully differentiated between *C*Las-infected and healthy *D*. *citri*

**Figure 3:**
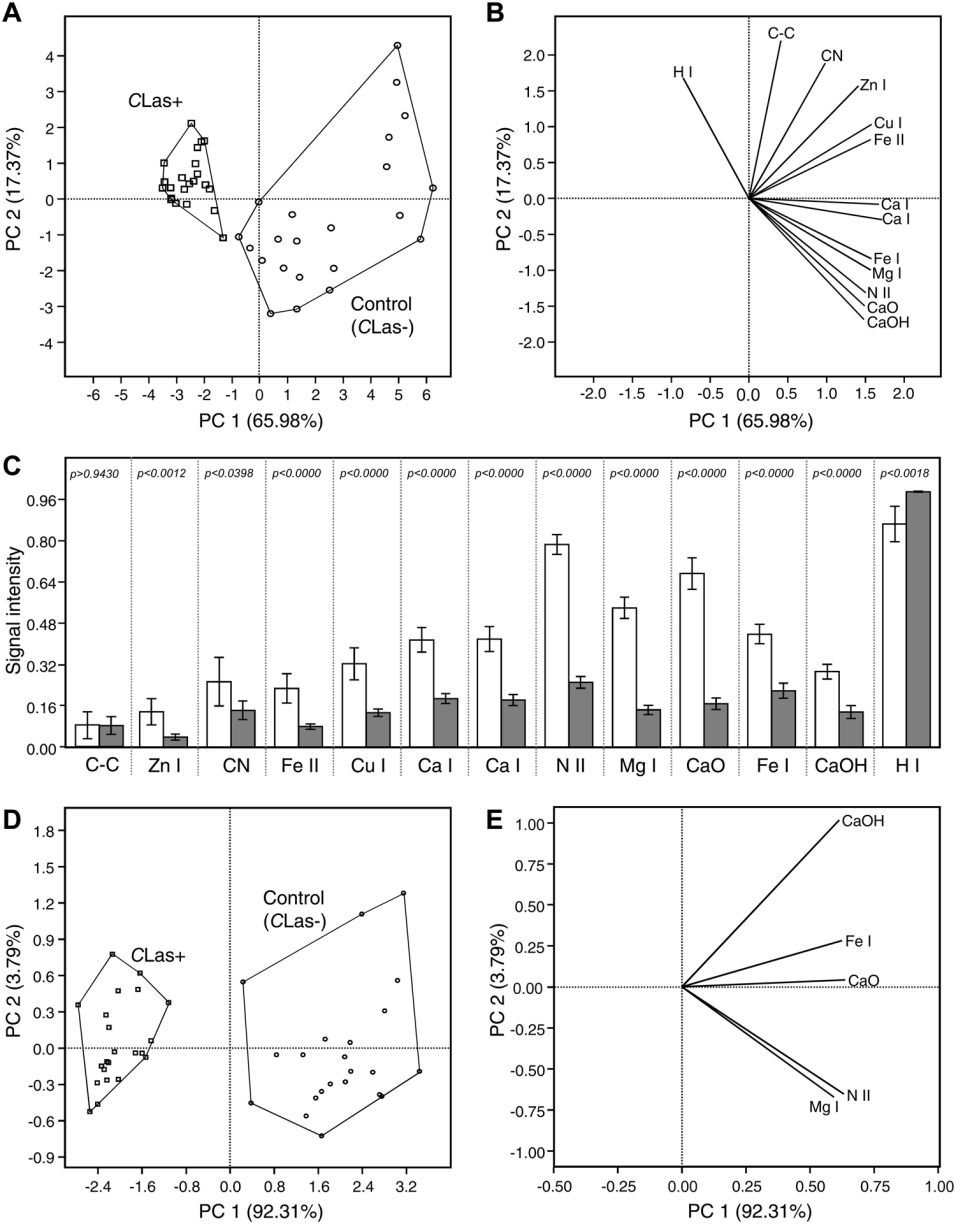
Differentiation between healthy and *C*Las-infected Asian citrus psyllid using elements identified by LIBS. (**A**) Principal component analysis of all identified elements (n = 22). (**B**) PCA-loading-plot for all identified elements. (**C**) Signal intensity of all elements identified by LIBS in healthy and *C*Las-infected Asian citrus psyllid. (**D**) Principal component analysis of five significant identified elements (n = 22). (**E**) PCA-loading-plot for five significant identified elements identified elements. Bars represent standard errors. *P*-values were calculated using the Student’s *t*-test.

The principal component analysis (PCA) generated using the normalized intensity of the thirteen detected peaks is shown in Fig. 3A,B. PC1 and PC2 accounted about 80% of the variation (Fig. 3A). As shown in the score plot (Fig. 3A), the *C*Las-infected *D*. *citri* were separated from the healthy *D*. *citri*, indicating that their elemental profile was different from that of healthy psyllids. The *C*Las-infected *D*. *citri* clustered in the left side of the score plot, whereas the healthy psyllids clustered to the right of the plot. The loading plot (Fig. 3B) showed that most of the detected peaks were higher in the control psyllids (first and fourth quadrants). The loading plot also showed that H I (656.2 nm), and Mg I (279.5 nm), were not important in the model because they lay in between the two groups (Fig. 3B).

### The Student’s *t*-test *C*Las-infected *D*. *citri* adults were deficient in most detected elements

When the intensities of the detected peaks were compared between the *C*Las-infected and healthy *D*. *citri* we found many differences. The C-C I (279.5 nm) peak was not significantly different (*P* > 0.9430) between the *C*Las-infected and healthy psyllids (Fig. 3C). The intensities of Fe II (at 298.9 nm), Zn I, C-N, and H I peak in *C*Las-infected psyllids shows small differences between healthy and *C*Las-infected psyllids (Fig. 3C). However, the intensity of Fe I (566.3 nm), Cu I, Ca I (435.3 nm), Ca I (462.1 nm), N II, CaO band (547–556), and CaOH (610.2) peaks in *C*Las-infected psyllids were dramatically lower (*P* < 0.0000) than those of healthy psyllids (Fig. 3C). These results indicated that Fe I (566.3 nm), Ca I (435.3 nm), Ca I (462.1 nm), N II (499.9), C-C (516.2), CaO band (547–556), and CaOH (610.2) peaks were the best markers for differentiation between the *C*Las-infected and healthy psyllids. Furthermore, these results suggested that *C*Las-infected *D*. *citri* were deficient in zinc, iron, copper, magnesium, calcium, and nitrogen.

### Filtering the data showed that only five peaks were necessary to discriminate between healthy and *C*Las-infected psyllids

Using the results from Fig. 3C, we refined the PCA model by using only five peaks (N II, Mg I, CaO, Fe I, and CaOH) (Fig. 3D,E). The scatter plot generated using these five peaks showed better separation between *C*Las-infected and healthy psyllids (Fig. 3D), indicating that the eliminated peaks were not significant for the model. The *C*Las-infected psyllids clustered together in the left of the plot and were totally separated from the controls, which clustered together in the right side of the score plot (Fig. 3D). In this analysis, PC1 and PC2 accounted for about 96% of the variation (Fig. 3E). In agreement with the Student’s *t*-test, all five selected peaks (N II, C-C, CaO, Fe I, and CaOH) were significantly lower in *C*Las-infected psyllids (Fig. 3E). These selected peaks correlated with the control psyllids and appeared on the right side of the loading plot (Fig. 3E).

## Discussion

### Evaluation of the classification model

Thirteen peaks from seven different elements were detected in healthy and *C*Las-infected *D*. *citri*. We decided to implement PCA because it can efficiently identify outliers and has been successfully used for classification of LIBS data^34^. The PCA generated using all of the detected peaks showed the existence of two main clusters (healthy and *C*Las-infected *D*. *citri*). This result indicated that LIBS can be used to differentiate between *C*Las-infected and healthy psyllids. Previous studies on *D*. *citri* showed that *C*Las infection can produce a large number of nutritional changes in its host insect^35–38^.

It has also been shown that LIBS can be successfully used to differentiate between HLB-symptomatic and healthy citrus leaves^29^, but no clear separation was observed. Unfortunately, no PCR was performed in the previous study to confirm the presence of the *C*Las titer in the leaves. Twenty-nine peaks from nine different elements were identified in citrus leaves, however only thirteen peaks were found to useful for the multivariate analysis^29^. Because some elements such as hydrogen, oxygen, and nitrogen exist at high background levels in the ambient atmosphere, these elements were hard to measure in citrus as the LIBS was conducted at ambient conditions^29^. In a similar study, LIBS was also used to differentiate between *C*Las-infected and healthy citrus plants by analyzing the major macro- and micronutrients^28^. Analysis of the LIBS using soft independent modeling of class analogy (SIMCA) data was able to detect *C*Las-infected plants from the first month^28^.

The *t*-test showed that most of the differences between *C*Las-infected and healthy psyllids were observed in the following peaks: N II, CaO, Mg1, CaOH, and Fe I. Consequently, we eliminated the rest of the peaks and refined the PCA analysis using these five peaks which significantly improved the separation between the *C*Las-infected and healthy psyllids. This result showed that the reduced PCA model (5 peaks from four elements) can be successfully used to differentiate between *C*Las-infected and healthy psyllids. The reduction in the number of wavelengths required for the efficient classification of *C*Las-infected and healthy insects makes it possible to create a portable and low-cost instrument which does not require a spectrometer. This portable detector could be built using just a few selective filters.

### Nutritional Changes in *C*Las-infected *D*. *citri*

The LIBS compositional analysis showed that *C*Las-infected adult psyllids were low in iron, zinc, copper, magnesium, nitrogen, and calcium, indicating that *C*Las-infected psyllids were under nutritional stress. Previous studies on citrus plants showed that *C*Las infection can produce a large number of nutritional changes. Zinc, magnesium, iron, nitrogen, and phosphorus were lower in *C*Las-infected plants compared to healthy plants^39^. LIBS also showed that *C*Las-infected citrus plants were also deficient in magnesium, potassium, calcium, copper, silicon, sodium, and titanium^29^. In addition, aluminum, silicon, titanium, manganese, nickel, copper, zinc, rubidium, strontium, and zirconium were also present at low levels in *C*Las-infected ‘pineapple’ sweet orange juice^40^. It was also reported that *C*Las-infected citrus trees were deficient in phosphorus, indicating that phosphorus could be required for the growth of *C*Las^41^. The previous results together indicated that *C*Las could acquires these elements from its host. In fact, many researchers believe that *C*Las pathogenicity is due to nutrient depletion and energy parasitism^42^. Many elements such as iron, zinc, copper, and manganese could be essential for the growth of *C*Las because they act as cofactors for various essential enzymes. The presence of the *znuABC* genes, which are responsible for the import of zinc, indicated that zinc was an essential element for *C*Las^43^. They suggested that the uptake of zinc by *C*Las from its host plant results in zinc deficiency. The reduction in micro and macronutrients in *C*Las-infected citrus may also result from root damage. It is believed that plugging of the phloem by *C*Las could stop the circulation of the phloem sap from the leaves (source) to the roots (sink), which may compromise root function, decrease root mass, lower the ability to absorb water and minerals, and ultimately lead to tree death^29^.

This study showed that LIBS can successfully measure various elements in the haemolymph of small insects like *D*. *citri*. This method is fast (less than 5 min/insect) and does not require sample preparation as in the case of inductively coupled plasma optical emission spectroscopy (ICP-OES). In addition, our results showed that LIBS can be performed on small samples (~0.01–1 µL) and enables simultaneous multi-elemental analysis.

## Conclusion

Herein we demonstrated that LIBS enables a fast, objective, and reliable diagnosis of the *C*Las pathogen in *D*. *citri* psyllids. The PCA analysis showed that LIBS could be successfully used to differentiate between *C*Las-infected and healthy *D*. *citri*. In addition, the *t*-test applied on the LIBS spectra provided insights about the elemental changes in *C*Las-infected psyllids. Considering the great separation between the *C*Las-infected and healthy psyllids, our results suggested that LIBS could be used for rapid screening of *C*Las in *D*. *citri*. Finally, LIBS could also be extended to study other plant insect-borne diseases such as citrus tristeza virus and potato zebra chip disease as well as human insect-borne diseases such as zika and malaria.
